# Isotopic composition of oceanic dissolved black carbon reveals non-riverine source

**DOI:** 10.1038/s41467-019-13111-7

**Published:** 2019-11-07

**Authors:** Sasha Wagner, Jay Brandes, Robert G. M. Spencer, Kun Ma, Sarah Z. Rosengard, Jose Mauro S. Moura, Aron Stubbins

**Affiliations:** 1 Department of Earth and Environmental Sciences, Rensselaer Polytechnic Institute, Troy, NY USA; 2 Department of Marine Sciences, Skidaway Institute of Oceanography, University of Georgia, Savannah, GA USA; 3 Department of Earth, Ocean, and Atmospheric Science, Florida State University, Tallahassee, FL USA; 4 Department of Earth, Ocean, and Atmospheric Sciences, University of British Columbia, Vancouver, BC Canada; 5 Center of Interdisciplinary Formation, Federal University of Western Para (UFOPA), Santarem, Para Brazil; 6 Departments of Marine and Environmental Sciences, Civil and Environmental Engineering, and Chemistry and Chemical Biology, Northeastern University, Boston, MA USA

**Keywords:** Carbon cycle, Marine chemistry, Limnology, Marine chemistry

## Abstract

A portion of the charcoal and soot produced during combustion processes on land (e.g., wildfire, burning of fossil fuels) enters aquatic systems as dissolved black carbon (DBC). In terms of mass flux, rivers are the main identified source of DBC to the oceans. Since DBC is believed to be representative of the refractory carbon pool, constraining sources of marine DBC is key to understanding the long-term persistence of carbon in our global oceans. Here, we use compound-specific stable carbon isotopes (δ^13^C) to reveal that DBC in the oceans is ~6‰ enriched in ^13^C compared to DBC exported by major rivers. This isotopic discrepancy indicates most riverine DBC is sequestered and/or rapidly degraded before it reaches the open ocean. Thus, we suggest that oceanic DBC does not predominantly originate from rivers and instead may be derived from another source with an isotopic signature similar to that of marine phytoplankton.

## Abstract (web summary)

Rivers are thought to be the largest source of the recalcitrant and abundant black carbon in the ocean. Here, Wagner and colleagues find distinct pools of black carbon between rivers and the open ocean, challenging the long-held assumption that marine black carbon is of terrestrial origin.

## Introduction

Fire regimes are increasingly influenced by human activity and anthropogenic climate change^1^. The incomplete combustion of organic matter during vegetation fires and the burning of fossil fuels generates fire-derived or pyrogenic carbon, here referred to as black carbon (BC). Vegetation fires are the predominant source of terrestrial BC (~260 Tg-C yr^−1^)^2,3^ where it is estimated that >90% of the BC produced remains on-site^3,4^. A relatively small portion of terrestrial BC is emitted to the atmosphere as pyrogenic aerosols (8–17 Tg-C yr^−1^) which are primarily derived from fossil fuel combustion^5^. Regardless of its formation conditions, BC is carbon-rich compared to its unburned organic precursor material^6^. Charring results in the thermal condensation of biomolecular structures, thereby altering the reactivity of the precursor organic material. Thermal alteration of biomass increases its recalcitrance, leading to longer environmental residence times which has implications when including BC in global carbon budgets^5,7^. BC deposited on the landscape becomes incorporated into soils to comprise ~14% of soil organic carbon stocks globally^8^. Soil BC is then mobilized to inland waters via wind, erosion, and/or leaching processes^9^, serving as a continuous source of fire-derived carbon to aquatic systems^10^. In natural waters, BC occurs in both dissolved and particulate forms^10^ and factors controlling the lateral transport of soil BC to inland waters are still being refined^9^. However, current estimates suggest roughly half of total BC entering waterways occurs as dissolved BC (DBC)^11,12^.

DBC is one of the most prevalent organic molecular classes that has been quantified in the global ocean, where it occurs at concentrations between 600 and 810 nM-C, equivalent to ~2% of total DOC^13,14^. In terms of mass flux, rivers are the largest identified source of DBC to the ocean^10^. The annual flux of riverine DBC to the oceans (27 Tg-C yr^−1^)^11^ would replace the standing stock of oceanic DBC (14 Pg-C)^13,14^ within ~500 years. This estimate is considerably shorter than the apparent age of DBC measured in either the surface or deep ocean (4800 and 23,000 ^14^C years, respectively)^14^. Thus, assuming riverine DBC is of near-modern age^14,15^, significant removal of riverine DBC needs to occur in order for flux- and ^14^C-derived turnover times to match, even if rivers were the only source of oceanic DBC. The same discrepancy has been noted for bulk DOC, with riverine inputs capable of replacing the entire oceanic DOC pool within ~2600 years, yet radiocarbon analyses suggest the average age of oceanic DOC is 4000–6000 ^14^C years^16–18^. Although riverine DOC could replace the oceanic DOC pool approximately two times over, biomarker and stable carbon isotope data indicate that oceanic DOC contains minimal amounts of terrestrial DOC and is instead dominated by autochthonous, marine DOC^16,19^. Stable carbon isotopic signatures of humics isolated from oceanic DOC are also consistent with a marine source^20^, further supporting the conclusion that terrestrial DOC is efficiently removed from the ocean. Compared to previous research on bulk DOC, isotopic studies of DBC are few. Previously reported DBC δ^13^C values include only one coastal sample (~−24‰) and two river samples (~−29‰)^21^. Although these data were limited, the observed isotopic discrepancy suggested coastal DBC may have a different source than, or be a degraded (altered) version of riverine DBC and raised the question as to whether this was a local or global phenomenon.

**Fig. 1:**
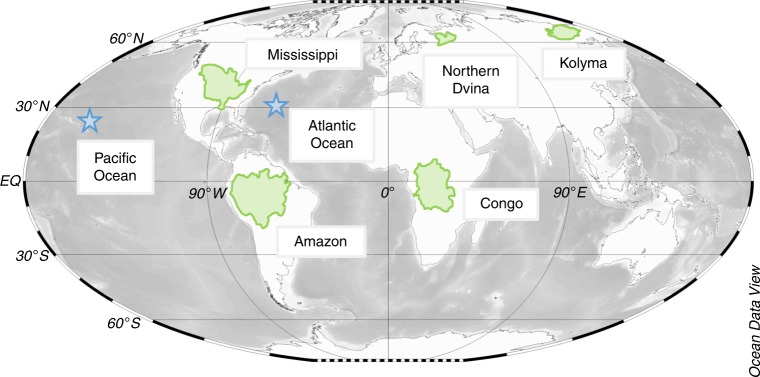
Map showing sites sampled for the current study. Green shaded regions denote river catchment margins. Blue stars indicates Atlantic and Pacific Ocean sampling locations. Base map was drawn using the Ocean Data View software package^72^

Thus, in the current study, we used compound-specific δ^13^C to probe the persistence of riverine DBC in the open ocean within a globally relevant sample set. If rivers were not the primary source of DBC in the ocean, then we would expect the δ^13^C signature of oceanic DBC to be different from that of rivers (~−29‰)^21^. If the null hypothesis was upheld, then oceanic DBC would exhibit δ^13^C signatures similar to that of riverine DBC. To test this hypothesis, water samples were collected from five major world rivers (Amazon, Congo, Northern Dvina, Kolyma, and Mississippi; total *n* = 12) and from the Pacific (*n* = 5) and Atlantic Oceans (*n* = 4) (Fig. 1). The sampled rivers deliver ~24% of freshwater discharge, ~17% of DOC^17^ and therefore ~17% of DBC^11^ to the oceans. The rivers vary in their DOC composition, land use, catchment area, geographical location, and climate, thereby capturing a wide range of potential terrestrial DBC sources and isotopic signatures^22–26^. Ocean samples were chosen to capture globally representative water masses and gradients in bulk DOC quality and source^16^. DOC was isolated by solid phase extraction (SPE-DOC)^27^. DBC was then measured in SPE-DOC by oxidizing condensed aromatics to individual benzenehexacarboxylic acid (B6CA) and benzenepentacarboxylic acid (B5CA) products^28^. Molecular markers B6CA and B5CA are the most dominant benzenepolycarboxylic acids (BPCAs) and are presumed to be exclusively of pyrogenic origin^29^. Individual BPCAs were then resolved, quantified, and isotopically characterized (BPCA-specific δ^13^C) to determine DBC δ^13^C values^21^. These data are presented and used to test the hypothesis that DBC found in the oceans is not isotopically consistent with DBC from rivers.

## Results and discussion

### Riverine and oceanic DBC stable carbon isotopic signatures

**Fig. 2:**
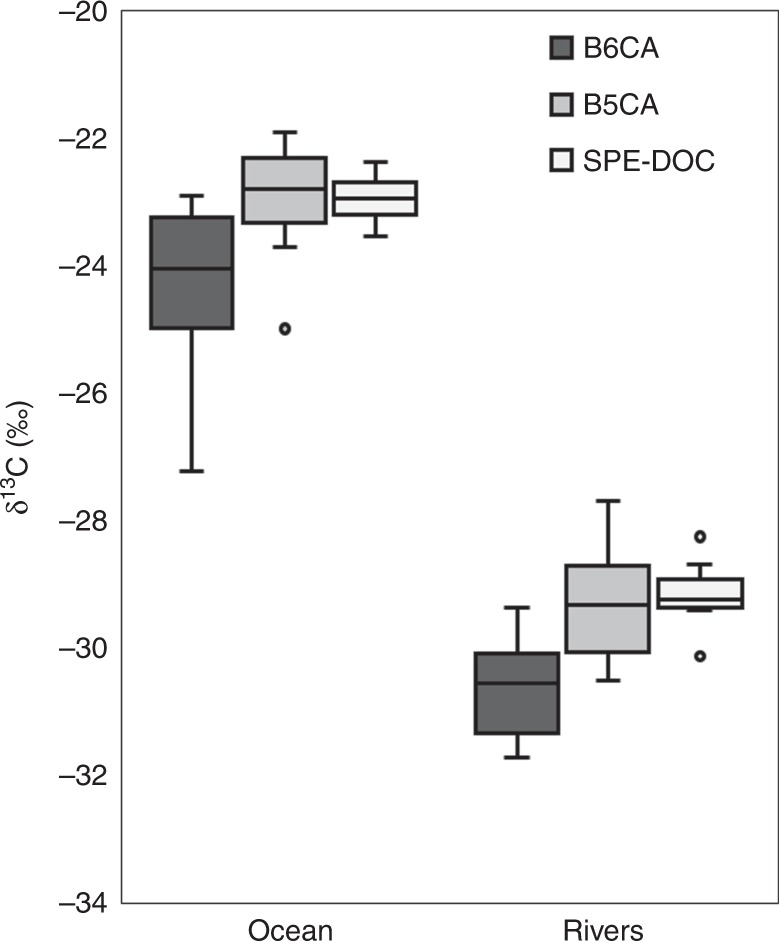
Riverine and oceanic stable carbon isotopic signatures. Box plots show the maximum value, minimum value, median value, interquartile range, and outliers for benzenehexacarboxylic acid (B6CA), benzenepentacarboxylic acid (B5CA), and dissolved organic carbon (SPE-DOC). The stable carbon isotopic (δ^13^C) values are relative to Vienna Pee Dee Belemnite (VPDB). Source data are provided as a Source Data file

The DOC and DBC concentrations captured by our riverine sample set varied by an order of magnitude (247–1310 and 19–115 μM-C, respectively; Table 1), spanning the range observed for large fluvial systems globally^11^. On average, riverine B6CA and B5CA were depleted in ^13^C and similar in isotopic composition to SPE-DOC (Table 1; Fig. 2), consistent with both DBC and DOC in rivers deriving from the same source^2,7^. Mean δ^13^C values for riverine B6CA and B5CA were −30.77 ± 1.51‰ and −29.44 ± 2.00‰, respectively. BPCA-specific δ^13^C values did not significantly vary among rivers (B6CA, *p* = 0.30; B5CA, *p* = 0.48; Table 1). However, SPE-DOC δ^13^C values were statistically different among some rivers (*p* = 0.0004). The significant result for SPE-DOC δ^13^C values and lack thereof for BPCA-specific δ^13^C values may have been driven by the increased precision of bulk (derived from elemental analysis) versus compound-specific (derived from liquid chromatography) isotopic measurements (Table 1). As previously observed for bulk DOC^30^, variations in SPE-DOC δ^13^C values among rivers could also be explained, in part, by considering the types of vegetation which serve as the source of DOC in each watershed. The δ^13^C composition of plants depends on its photosynthetic pathway for fixing atmospheric carbon. Plants utilizing the globally dominant C3 pathway (e.g., woody vegetation and most vascular plants) results in biomass that is relatively depleted in ^13^C (−22‰ to −30‰), whereas plants fixing carbon following the C4 pathways (e.g., corn, sugarcane, and some grasses) results in biomass that is more ^13^C-enriched (−10‰ to −14‰)^31,32^. The rainforest-dominated Amazon River catchment exported SPE-DOC that was most depleted in ^13^C, whereas the Mississippi, with increased proportions of grassland and agriculture^33^, exported SPE-DOC that was ~2‰ more enriched in ^13^C (Table 1). The Congo River watershed is roughly half forested and half grassland^34^, yielding intermediate SPE-DOC δ^13^C values (Table 1). SPE-DOC exported by the Northern Dvina River in the Arctic, which primarily drains peat lowlands^35^, had statistically similar δ^13^C values as the equatorial Congo River SPE-DOC (*p* = 0.09; Table 1). Although the reason for this was not specifically investigated, isotopic similarities between the Northern Dvina and Congo suggests SPE-DOC in each river derives from a ^13^C-depleted terrestrial organic matter source. Since differences in discharge could drive variations in DBC isotopic composition, we also compared multiple samples collected across the hydrograph from the Congo and Northern Dvina Rivers. The Congo River is a low latitude, tropical river with a relatively stable hydrological regime^36^. In contrast, the high latitude Northern Dvina River exhibits strong seasonal hydrology, where discharge peaks during the spring freshet and is accompanied by high DOC concentrations^24^. Throughout the sampling period, concentrations of DOC and DBC increased by a factor of two in the Congo River and by a factor of three in the Northern Dvina River (Table 1). However, δ^13^C values for SPE-DOC, B6CA, and B5CA were remarkably consistent (SD < 1‰; Table 1). These results suggest that the isotopic composition of DBC derived from global rivers is relatively constant, remaining consistently depleted in ^13^C regardless of land cover or hydrologic regime, and indicates that DBC is derived from similar source vegetation as SPE-DOC. Thus, the δ^13^C signature of BPCAs in rivers appear to be a faithful proxy for the source vegetation that presumably burnt to yield the DBC.

**Table 1:** Concentrations, qualitative ratios, and stable carbon isotopic values (δ^13^C) of DOC, DBC, and BPCA molecular markers in rivers the ocean

Site	Depth (m) or Discharge (m^3^ s^−^^1^)	DOC (µM-C)	DBC (µM-C)	DBC:DOC (%)	B6CA:B5CA	δ^13^C B6CA (‰)	δ^13^C B5CA (‰)	δ^13^C SPE DOC (‰)
Pacific	5	81	0.62	0.8	0.23	−24.89 ± 0.66	−22.26 ± 0.97	−23.32 ± 0.08
Pacific	110	76	0.63	0.8	0.24	−27.23 ± 0.46	−22.29 ± 0.45	−23.52 ± 0.08
Pacific	765	45	0.62	1.4	0.29	−25.03 ± 0.45	−22.29 ± 0.45	−22.88 ± 0.08
Pacific	1000	43	0.58	1.4	0.31	−24.50 ± 0.45	−21.86 ± 0.45	−22.92 ± 0.08
Pacific	3500	42	0.61	1.5	0.30	−24.03 ± 0.45	−22.77 ± 0.73	−22.92 ± 0.08
Atlantic	1	63	0.78	1.2	0.24	−23.59 ± 0.44	−22.93 ± 0.72	−23.02 ± 0.03
Atlantic	70	56	0.78	1.4	0.26	−23.32 ± 0.44	−22.89 ± 0.72	−22.85 ± 0.06
Atlantic	2000	44	0.75	1.7	0.30	−22.87 ± 0.79	−24.97 ± 0.72	−22.33 ± 0.04
Atlantic	3000	40	0.71	1.8	0.31	−23.10 ± 0.62	−23.67 ± 1.09	−22.44 ± 0.05
Amazon	N.D.	331	25	7.6	0.42	−31.77 ± 0.45	−29.42 ± 0.45	−30.17 ± 0.08
Congo	23,740	540	37	6.8	0.49	−30.27 ± 0.44	−29.45 ± 0.72	−29.38 ± 0.08
Congo	43,940	886	69	7.8	0.49	−30.52 ± 0.44	−29.30 ± 0.72	−29.34 ± 0.08
Congo	54,120	1007	76	7.6	0.50	−30.98 ± 0.44	−29.93 ± 0.72	−29.44 ± 0.08
Congo	41,810	729	59	8.2	0.50	−30.47 ± 0.44	−29.05 ± 0.72	−29.29 ± 0.08
Congo	32,000	545	46	8.4	0.49	−30.06 ± 0.44	−28.62 ± 0.72	−29.13 ± 0.08
N. Dvina	1610	519	42	8.0	0.57	−31.43 ± 0.77	−30.53 ± 0.69	−28.94 ± 0.08
N. Dvina	637	349	29	8.4	0.55	−30.67 ± 0.22	−29.16 ± 0.30	−28.70 ± 0.08
N. Dvina	9287	1310	104	7.9	0.65	−31.61 ± 0.17	−30.54 ± 0.23	−29.26 ± 0.08
N. Dvina	13,380	1303	115	8.9	0.70	−31.27 ± 0.23	−30.16 ± 0.42	−29.15 ± 0.08
Kolyma	N.D.	327	20	6.2	0.43	−30.01 ± 0.45	−27.92 ± 0.45	N.D.
Mississippi	N.D.	247	18	7.3	0.30	−29.40 ± 0.45	−27.70 ± 0.45	−28.27 ± 0.01

Depths are listed for oceanic samples and discharge values are listed for the Congo and Northern Dvina River samples. The δ^13^C values are relative to Vienna Pee Dee Belemnite (VPDB)

**Fig. 3:**
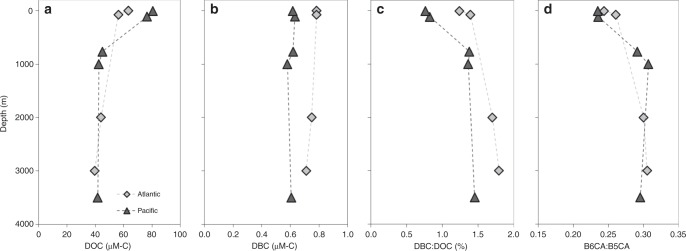
Atlantic and Pacific Ocean depth trends. Depth versus **a** dissolved organic carbon (DOC) concentration, **b** dissolved black carbon (DBC) concentration, **c** DBC:DOC ratio, and **d** ratio of benzenehexacarboxylic acid to pentacarboxylic acid (B6CA:B5CA) are shown for samples collected at Atlantic Ocean (light grey diamonds) and Pacific Ocean (dark grey triangles) sites. Source data are provided as a Source Data file

At both the Atlantic and Pacific Ocean sites, DOC concentrations were highest in surface waters (63 and 81 μM-C, respectively), reached minima at depth (≥1000 m; ~42 μM-C; Fig. 3a), and were in line with profiles previously reported for these locations^18^. SPE-DOC δ^13^C values were similar among oceanic samples (average = −22.9 ± 0.4‰; Fig. 2; Table 1) and consistent with oceanic DOC being derived from a marine phytoplankton source^16^. The majority of DOC released from phytoplankton is termed labile as it is rapidly degraded before it can accumulate^37^. DOC that persists for weeks to years is termed semilabile and is observationally defined as the pool of DOC which accumulates in surface waters, but does not survive over the longer timeframes required to mix into the deep ocean^18^. Semilabile DOC is enriched in hydrolysable sugars and other products of recent biological production^38^, but depleted in aromatic components^13^. It is this semilabile DOC which contributes to the DBC-depleted, DOC-enriched surface waters observed here (Fig. 3a, c). In the deep ocean (>1000 m depth), DOC concentrations are low^18^ (Fig. 3a) and ^14^C-depleted relative to DOC in surface waters^16^. Thus, DOC in the deep ocean is termed refractory as it appears to persist for thousands of years, becoming mixed throughout the global ocean^18^. In contrast to bulk DOC, DBC concentrations did not vary with depth (Fig. 3b), consistent with the apparent refractory nature and long-term stability of DBC in the ocean^14^. The refractory DOC pool is one of the largest organic carbon stores on Earth and the largest in the ocean^18^, thus investigating its persistence is of major scientific interest and societal importance^37^. Since DBC appears to be representative of this refractory DOC^13^, a critical step in this endeavor is to constrain sources of oceanic DBC.

In contrast to bulk DOC, DBC concentrations did not vary with depth (Fig. 3b), but were higher, on average, in the Atlantic (0.76 ± 0.03 μM-C) than in the Pacific Ocean (0.61 ± 0.02 μM-C; *p* = 0.0001; Table 1). The Atlantic Ocean constitutes 24% of the global ocean volume but receives half of global freshwater discharge, while the Pacific Ocean constitutes 51% of the total ocean and receives only 20% of global freshwater discharge^39^. Lignin, an unambiguous molecular tracer for higher plant-derived material and terrestrial DOC, is also higher in the Atlantic Ocean compared to the Pacific Ocean^40–42^. Higher concentrations of lignin in the Atlantic Ocean have been ascribed to the greater relative proportion of river water received by this ocean basin^40–42^. Concentrations of DBC in rivers were roughly two orders of magnitude higher than in the ocean (Table 1). Consistent with previous studies^11,14,43^, DBC in rivers constituted a greater proportion of bulk DOC (6–9%) and exhibited a more condensed aromatic signature (higher B6CA:B5CA) than in the ocean samples, where DBC comprised <2% of DOC and had lower B6CA:B5CA values (Table 1).

**Fig. 4:**
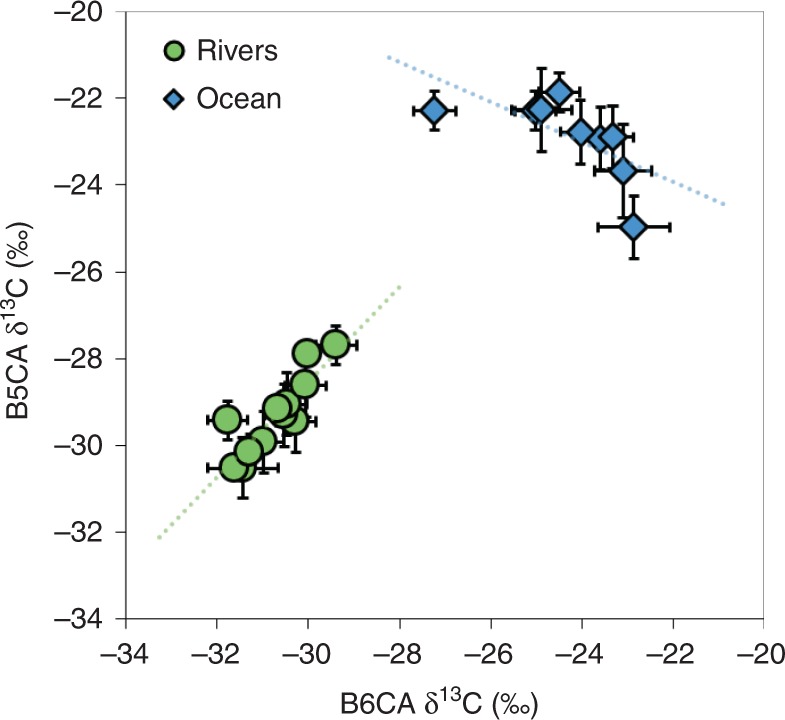
Relationship between compound-specific isotopic values. The stable carbon isotopic composition (δ^13^C) of benzenepentacarboxylic acid (B5CA) was plotted against that of benzenehexacarboxylic acid (B6CA) for samples collected from rivers (green circles) and the ocean (blue diamonds). Linear regressions are denoted by dotted lines and were B5CA δ^13^C = 1.09 × B6CA δ^13^C + 4.21 (*R*^2^ = 0.74; *p* = 0.0003) for riverine samples and B5CA δ^13^C = −0.46 × B6CA δ^13^C − 34.05 (*R*^2^ = 0.43; *p* > 0.05) for oceanic samples. Error bars represent propagated error associated with isotopic calibration or replicate error (1 SD), whichever is larger. The δ^13^C values are relative to Vienna Pee Dee Belemnite (VPDB). Source data are provided as a Source Data file

BPCA-specific δ^13^C values revealed a stark contrast in the isotopic composition of DBC in global rivers and the ocean (Fig. 2). Mean δ^13^C values for ocean B6CA and B5CA were −24.28 ± 1.62‰ and −22.88 ± 2.19‰, respectively. Taken together, mean BPCA-specific δ^13^C values were statistically similar between Atlantic and Pacific Ocean sites (B6CA, *p* = 0.20; B5CA, *p* = 0.48; Table 1). On average, BPCAs derived from oceanic DBC were significantly more enriched in ^13^C (by ~6‰) than BPCAs derived from riverine DBC (*p* < 0.0001; Fig. 2). In rivers, B5CA- and B6CA-specific δ^13^C values were significantly correlated (*R*^2^ = 0.74, *p* = 0.0003) and B5CA was consistently more enriched in ^13^C than B6CA (Fig. 4). The isotopic offset between B5CA and B6CA in rivers ranged from 0.82 to 2.35‰ (Table 1). In contrast, δ^13^C values for oceanic B5CA and B6CA were not linearly related (*R*^2^ = 0.43, *p* > 0.05; Fig. 4), and the isotopic offset between B5CA and B6CA ranged from −2.11 to 4.95‰ (Table 1). The correlation between B5CA and B6CA δ^13^C values in rivers was expected, since we presume both molecular markers to be derived from the same pool of fire-derived condensed aromatics originating from soil BC. The observed difference between B5CA and B6CA δ^13^C values could be an intrinsic property of riverine DBC, thus providing further support for non-riverine sources of oceanic DBC. However, further research is required to elucidate specific biogeochemical mechanisms which control BPCA δ^13^C offset values in terrestrial and marine environments. Based upon our current results, hydrology and watershed land cover accounted for ~2‰ variability in riverine endmember DBC δ^13^C values (Table 1; Fig. 2). Oceanic BPCA-specific δ^13^C signatures were generally conserved with depth (Table 1), however B6CA was significantly depleted in ^13^C (~2‰ lower) at 110 m depth in the Pacific Ocean (*p* < 0.01) and B5CA was significantly depleted in ^13^C (~2‰ lower) at 2000 m depth in the Atlantic Ocean (*p* < 0.05). Overall, BPCA-specific δ^13^C values for different ocean water masses varied by only ~2‰ (Table 1; Fig. 2). Thus, endmember variability alone cannot account for the large isotopic discrepancy observed between BPCAs in rivers and the ocean. As such, our results indicate a non-riverine source for DBC quantified in the open ocean.

### Riverine and oceanic DBC isotopic discrepancy implications

The application of BPCA-specific δ^13^C analysis for constraining sources of oceanic DBC assumes riverine BPCA-specific δ^13^C values are not fractionated (altered) as DBC transits coastal waters. Photochemical degradation has been shown to alter and remove DBC in natural waters^44,45^. Exposure to sunlight results in a photochemically processed DOC pool that is relatively enriched in ^13^C^46,47^, due to the preferential loss of aromatic, ^13^C-depleted DOC compound classes (e.g., lignin^48^). However, BPCA-specific isotopic analyses bypass the molecular heterogeneity of bulk DOC to target individual compound classes (e.g., condensed aromatics^21^). Therefore, exposure of DBC to sunlight is expected to degrade condensed aromatics similarly, where one carbon isotope is not preferentially photomineralized over another (^12^C versus ^13^C) within the same compound class. In the current study, lower DBC:DOC and B6CA:B5CA values observed for shallow ocean depths (Fig. 3c, d) is consistent with photodegradation of DBC in sunlit surface waters^44^. BPCA-specific δ^13^C signatures in sun-bleached surface waters and the dark abyssal ocean were statistically identical (Table 1; *p* > 0.05), suggesting photochemistry has minimal-to-no impact upon DBC δ^13^C values. However, the impact of sun exposure upon riverine BPCA-specific δ^13^C signatures must be directly tested to confirm isotopic stability during photodegradation of DBC. If the physical removal of DBC from the water column (e.g., sorption to sinking particles^49^), was primarily responsible for the observed offset between riverine and oceanic DBC δ^13^C values, then we would expect a gradient in δ^13^C values with depth in the ocean. However, the δ^13^C signature of oceanic DBC was fairly consistent with depth (Table 1). We look to Δ^14^C assessments for further insight on marine DBC cycling^14,43^. The apparent radiocarbon age of BPCAs increases in the deep ocean, suggesting the deep oceanic DBC pool is older (up to 23,000 ^14^C-years) and more refractory than the younger DBC (4,800 ^14^C-years) in the surface ocean^14^, mirroring trends observed for bulk DOC in the oceans^18^. The relative consistency of DBC δ^13^C signatures down-profile and similarity of BPCA-specific δ^13^C values between Atlantic and Pacific Ocean sites (Table 1) suggests a common source for modern DBC in the surface ocean and ancient DBC in the abyssal ocean. Assuming degradation processes do not result in a ^13^C-enriched DBC pool, then isotopic trends for DBC match those for bulk DOC, where δ^13^C signatures indicate oceanic DOC to be dominated by marine sources with minimal contributions from terrestrial DOC^15,20,50^.

Since our data are inconsistent with a riverine source for oceanic DBC (Fig. 2), we consider other sources of BPCAs to the open ocean. BPCA-specific δ^13^C values are similar to SPE-DOC δ^13^C values (Table 1), phytoplankton biomass, bulk marine DOC, and marine particulate organic carbon^16^. Based upon the isotopic signatures alone, a simple hypothesis is that the carbon in DBC, like bulk DOC, was originally fixed by phytoplanktonic production in the surface ocean. However, evidence for the autochthonous production of DBC in ocean waters does not exist and remains an area open for investigation. Biotic processes are proposed to alter the chemistry or diversity of freshly produced phytoplanktonic DOC, eventually resulting in the refractory pool of DOC observed in the ocean^51^. However, specific mechanisms which control the composition and reactivity of molecules which comprise the oceanic refractory DOC pool remain elusive^52^. Abiotic processes alter DOC, and likely also DBC, in the ocean. For instance, BPCAs could derive from organic material thermally processed or released by hydrothermal vents^53^. Hydrothermal vent conditions have also been shown to degrade refractory DOC in seawater^54^. Therefore, the net effect of these geothermally active hot spots on deep ocean DBC should be further investigated. The highly condensed (graphitic) BC contained in marine sediments is ancient (has a fossil Δ^14^C value), terrestrial in origin, and has a marine-like δ^13^C signature (−19 to −21‰), consistent with BC derived from bedrock erosion on land^55^. Petrogenic organic carbon constitutes ~20% of riverine particulate organic carbon exported globally^56^ and has been shown to produce BPCAs upon oxidation^57^. Therefore, the dissolution of petrogenic BC from sinking particles or resuspended marine sediments could be another, yet unidentified, source of ^14^C-depleted, ^13^C-enriched BPCAs in the abyssal ocean. The derivation of BPCAs from petrogenic material suggests that B5CA and B6CA may not be exclusively representative of fire-derived organics^58^. The nitric acid reaction which converts organic matter to BPCAs is complex and lacks a systematic pattern for the oxidation of condensed aromatics^59^. Thus, nonpyrogenic sources of marine DBC may be possible, but remain unproven.

Non-riverine, allochthonous sources of condensed organics which align with observed isotopic signatures may also contribute to the marine DBC pool. For instance, the atmospheric deposition of combustion-derived aerosols has recently been identified as the second-largest flux of DBC to marine surface waters (2 Tg-C per year)^60^. Globally, BC aerosols are derived roughly equally from both fossil fuel and biomass combustion processes^61^. However, contributions of fossil carbon to BC aerosols vary dramatically, ranging from ~10 to ~90% depending on geographic location and time of year^62–66^. While there is evidence that aerosol BC δ^13^C signatures are modulated by biomass type and combustion conditions, the isotopic composition of the charred precursor material is largely retained^67^. Global isotopic assessments of atmospheric BC do not exist, but δ^13^C values for BC aerosols over South Asia were shown to vary by up to ~4‰ and within the range observed for oceanic DBC^62^. In addition, BC in south Atlantic sediments exhibit a δ^13^C signature that is slightly enriched (−23 to −26‰)^68^ compared to riverine DBC measured here (Fig. 2). Based upon these limited data, it is possible that aerosol-derived DBC has enriched δ^13^C signatures that could balance the observed discrepancy between riverine and marine DBC pools. However, a globally representative sample set is needed to constrain the isotopic signature of oceanic DBC sourced from pyrogenic aerosols.

Rivers efficiently mobilize DBC from the land^11^, but mass balance estimates and our stable carbon isotopic signatures suggest most of this fire-derived material does not persist in the open ocean. Our findings are consistent with previous observations for the lack of bulk terrigenous dissolved organic matter in the ocean^19^. No information is available on the behavior of DBC as it transits estuarine and coastal zones, highlighting a critical area of research needed to fully understand connections between riverine and oceanic DBC pools. Further research is also needed to probe the stability of DBC δ^13^C signatures during photo-exposure and other processes, including sorption/desorption. Riverine export and atmospheric deposition deliver DBC to the ocean in excess of the apparent turnover of oceanic DBC^11,14,60^. Our isotopic data indicate additional, marine-derived sources are also likely. Thus, if BPCAs represent a biogeochemically coherent pool of compounds, then oceanic DBC turnover rates must be significantly faster than radiocarbon dating suggests^14^. Similarities between BPCA-specific and SPE-DOC δ^13^C values (Fig. 2) raise questions as to whether the BPCA method is capable of identifying sources of non-pyrogenic organic material. If δ^13^C fractionation during DBC removal processes is indeed negligible, then we will need to reexamine whether these condensed aromatic compounds truly represent the impact of fire upon the chemistry of the ocean, and search for new mechanisms which form this increasingly enigmatic molecular class.

## Methods

### Sample collection

Pacific Ocean samples were collected from Station ALOHA (22.45°N, 158.00°W) during the Hawaii Ocean Time-series (HOT) 301 cruise aboard the R/V *Ka`imikai-O-Kanaloa*. Samples from surface and mesopelagic depths (5, 110, 765, and 1000 m) were obtained from two casts on April 17, 2018 and the deep water sample (3500 m) was collected on April 18, 2018. Atlantic Ocean samples were collected from Hydrostation S (31.67°N, 64.17°W) during the Bermuda Atlantic Time Series (BATS) 358 cruise aboard the R/V *Atlantic Explorer*. Water samples were collected along a depth profile (1, 70, 2000, and 3000 m) on April 8, 2019. All seawater samples were collected using Niskin bottles mounted to the CTD rosette. For each sample, ~10 L was passed, by gravity, through a pre-cleaned in-line capsule filter (Whatman Polycap; 0.2 μm) into acid-cleaned Nalgene fluorinated HDPE carboys and pre-combusted glass vials and acidified to pH 2 using HCl prior to solid phase extraction and DOC analysis.

River samples were collected from the main stem of the Amazon, Congo, Northern Dvina, Kolyma, and Mississippi Rivers, upstream of the river mouth where marine inputs were not observed (salinity = 0). The Amazon River is the largest river on Earth, drains the world’s largest tropical rainforest, and represents a major link in the transfer of carbon between land and sea^69,70^. One surface water sample was collected from the Amazon River near Óbidos (Brazil; April 20, 2018; 1.92°S, 55.53°W). The Congo River, located in equatorial Africa, is the second largest river on Earth^23,34^ and was sampled from a site upstream of the cities of Kinshasa–Brazzaville on the main stem (DR Congo; 4.18°S, 15.21°E). Five samples were collected from the Congo River between August 2011 and April 2012, where discharge ranged from 23,740 to 54,120 m^3^ s^−1^, to account for potential hydrologically driven variations in DBC isotopic composition in a low latitude, subtropical river. The Northern Dvina River watershed is located in northwestern Russia, primarily drains low relief peatlands, is not underlain by permafrost^35^, and is a major contributor to pan-Arctic terrestrial DOC fluxes^24^. Four samples were collected from the Northern Dvina River in Arkhangelsk (64.55°N, 40.51°E) between October 2013 and May 2016, where discharge ranged from 637 to 13,380 m^3^ s^−1^, to capture isotopic variations in a high latitude river with extremely variable hydrology. The Kolyma River discharges into the Arctic Ocean and is a large, northern high-latitude river system that is, in contrast to the Northern Dvina, completely underlain by continuous permafrost^25,35^. One sample was collected from the Kolyma River, upstream of the city of Cherskiy (August 30, 2015; 68.75°N, 161.29°E). The Mississippi River drainage basin spans ~40% of the conterminous United States and contains one of the most productive agricultural regions in the world^26,33^. One sample was collected from the Belle Chase Ferry Landing on the Mississippi River, just south of New Orleans, LA (USA; February 23, 2016; 29.82°N, 90.00°W). All river samples were collected in situ using an oil-free pump equipped with a pre-cleaned, in-line capsule filter (Whatman Polycap, 0.2 μm) directly into acid-cleaned Nalgene bottles and pre-combusted glass vials. Samples were acidified to pH 2 using HCl prior to solid phase extraction and DOC analysis.

### DOC analysis and solid phase extraction

Filtered and acidified samples were analyzed for DOC, measured as nonpurgable organic carbon using a Shimadzu TOC-L CPH analyzer equipped with an ASI-L autosampler. Sample DOC was quantified using a calibration curve made with a potassium hydrogen phthalate stock solution. Measurement accuracy and reproducibility was assessed by analyzing deep seawater and low carbon water reference materials obtained from the Consensus Reference Material (CRM) project (https://hansell-lab.rsmas.miami.edu/consensus-reference-material/index.html). Analyses of CRM were within 5% of reported values.

DOC was isolated from samples via solid phase extraction (SPE; Varian Bond Elut PPL cartridges, 1 g, 6 mL)^27^ prior to DBC analysis. Briefly, SPE cartridges were conditioned with methanol, ultrapure water, and acidified water. Filtered and acidified samples were passed through the SPE cartridges by gravity. Isolated DOC was then eluted from the SPE cartridges with methanol and stored at −20 °C until DBC analysis. Recovery of DOC by SPE was 74% for riverine and 48% for oceanic samples, on average (all data are provided in the Supplementary Dataset). DOC recoveries in the current study are consistent with those observed previously for fresh and marine waters^27^.

### DBC quantification

Sample DBC was quantified using the BPCA method, which chemically degrades condensed aromatic compounds into benzenehexacarboxylic acid (B6CA) and benzenepentacarboxylic acid (B5CA) molecular markers^28^. BPCAs were oxidized and quantified following previously described methods^21,28^. Briefly, aliquots of SPE-DOC (~0.5 mg-C equivalents) were transferred to 2 mL glass ampules and dried under a stream of argon until complete evaporation of methanol. Concentrated HNO_3_ (0.5 mL) was added to each ampule, then ampules were flame-sealed and heated to 160 °C for 6 h. After oxidation, ampules were opened and HNO_3_ was dried at 60 °C under a stream of argon. The BPCA-containing residue was re-dissolved in dilute H_3_PO_4_ for subsequent analysis by high performance liquid chromatography (HPLC). Quantification of BPCAs was performed using a Dionex Ultimate 3000 HPLC system equipped with an autosampler, pump, and diode array detector. B6CA and B5CA were separated on an Agilent Poroshell 120 phenyl-hexyl column (4.6 × 150 mm, 2.7 µm) using an aqueous gradient of H_3_PO_4_ (0.6 M; pH 1) and sodium phosphate (20 mM; pH 6) buffers^21^. BPCAs were quantified using calibration curves for commercially available B6CA and B5CA using a 5 mM BPCA-C stock solution. River samples were oxidized and analyzed in duplicate. Ocean samples were oxidized and analyzed in triplicate. The average coefficients of variation for replicate measurements of B6CA and B5CA were <5%. Sample DBC concentrations were calculated using the established power relationship between DBC (µM-C) and the sum of B6CA and B5CA (nM-BPCA) shown below (*n* = 352, *R* = 998, *p* < 0.0001): [DBC] = 0.0891 × ([B6CA + B5CA])^0.9175^^71^. DBC concentrations calculated using this equation are directly comparable to those measured in previous studies^10,11^.

### Stable carbon isotopic analyses

Riverine and oceanic SPE-DOC methanol extracts were transferred to smooth-walled tin capsules (~0.25 mg-C per capsule) and methanol evaporated to dryness in an oven set to 60 °C. Sample-containing tin capsules were folded and combusted using a Thermo Scientific Flash EA Isolink CNSOH interfaced with a Thermo Scientific Delta V Plus IRMS. The δ^13^C composition of each sample was calibrated against an internal lab organic matter reference material (chitin from shrimp shells), which was previously calibrated against NIST glutamic acid (RM 8573) and sucrose (RM 8542) primary isotope reference standards. The ^13^C content is expressed in δ^13^C per mil (‰) notation relative to Vienna Pee Dee Belemnite (VPDB). River samples were measured in duplicate and ocean samples were measured in triplicate. The standard deviation of replicate EA-IRMS measurements was <0.1‰.

Compound-specific stable carbon isotopic values for individual BPCAs were measured using a Dionex Ultimate 3000 HPLC connected to a Delta V IRMS via an LC Isolink interface following methods detailed previously^21^. Online oxidation quantitatively converts baseline-separated BPCAs to CO_2_. BPCA-derived CO_2_ is then extracted from the mobile phase and dried prior to detection by IRMS. The δ^13^C values for B5CA and B6CA standards were measured by EA-IRMS following the same procedure described above to calculate and correct for offsets in HPLC-IRMS δ^13^C measurements^21^. River samples were analyzed in duplicate and ocean samples were analyzed in triplicate. Standard deviations applied to corrected sample δ^13^C values were propagated to account for errors associated with replicate EA-IRMS standard BPCA measurements, HPLC-IRMS standard BPCA measurements, and HPLC-IRMS sample BPCA measurements. The ^13^C content is expressed in δ^13^C per mil (‰) notation relative to Vienna Pee Dee Belemnite (VPDB). The error associated with corrected δ^13^C values was typically <0.5‰.

### Statistical evaluations

Significant variations in BPCA-specific and SPE-DOC δ^13^C values among rivers were determined using a one-way ANOVA. Tukey’s HSD tests were used to probe whether two isotopic values were significantly different (e.g., Congo versus Northern Dvina River SPE-DOC δ^13^C values, comparing δ^13^C values between oceanic water masses). An unpaired *t* test was used to compare mean riverine and oceanic BPCA-specific and SPE-DOC δ^13^C values. The above-described statistical evaluations assume normal distributions among data subsets. Linear regressions were used to assess the relationship between B6CA- and B5CA-specific δ^13^C values in riverine and oceanic datasets.

### Reporting summary

Further information on research design is available in the Nature Research Reporting Summary linked to this article.

## Supplementary information

Supplementary Dataset

Reporting Summary

Source Data

## Data Availability

The authors declare that the data supporting the findings of this study are available within the paper and its Supplementary Dataset. The source data underlying Figs. 2–4 are provided in Table 1, the Supplementary Dataset, and the Source Data file.

## References

[1] Bowman, D. M. J. S. et al. Fire in the Earth system. *Science* **324**, 10.1126/science.1163886 (2009). 10.1126/science.1163886 19390038

[2] Santín, C. et al. Towards a global assessment of pyrogenic carbon from vegetation fires. *Global Change Biol*. **22**, 10.1111/gcb.12985 (2015). 10.1111/gcb.12985 26010729

[3] Jones MW, Santín C, van der Werf GR, Doerr SH (2019). Global fire emissions buffered by the production of pyrogenic carbon. Nat. Geosci..

[4] Kuhlbusch TAJ, Crutzen PJ (1995). Toward a global estimate of black carbon in residues of vegetation fires representing a sink of atmospheric CO_2_ and a source of O_2_. Glob. Biogeochem. Cycles.

[5] Bird MI, Wynn JG, Saiz G, Wurster CM, McBeath A (2015). The pyrogenic carbon cycle. Annu. Rev. Earth Planet. Sci..

[6] Schneider MPW, Hilf M, Vogt UF, Schmidt MWI (2010). The benzene polycarboxylic acid (BPCA) pattern of wood pyrolyzed between 200 °C and 1000 °C. Org. Geochem..

[7] Landry JS, Matthews HD (2017). The global pyrogenic carbon cycle and its impact on the level of atmospheric CO2 over past and future centuries. Glob. Change Biol..

[8] Reisser, M., Purves, R. S., Schmidt, M. W. I. & Abiven, S. Pyrogenic carbon in soils: a literature-based inventory and a global estimation of its content in soil organic carbon and stocks. *Front. Earth Sci*. **4**, 10.3389/feart.2016.00080 (2016).

[9] Abney, R. B. & Berhe, A. A. Pyrogenic carbon erosion: implications for stock and persistence of pyrogenic carbon in soil. *Front. Earth Sci*. **6**, 10.3389/feart.2018.00026 (2018).

[10] Wagner, S., Jaffé, R. & Stubbins, A. Dissolved black carbon in aquatic ecosystems. *Limnol. Oceanogr. Lett*. **3**, 10.1002/lol2.10076 (2018).

[11] Jaffé, R. et al. Global charcoal mobilization from soils via dissolution and riverine transport to the oceans. *Science* **340**, 10.1126/science.1231476 (2013). 10.1126/science.1231476 23599492

[12] Coppola AI (2018). Global-scale evidence for the refractory nature of riverine black carbon. Nat. Geosci..

[13] Dittmar T, Paeng J (2009). A heat-induced molecular signature in marine dissolved organic matter. Nat. Geosci..

[14] Coppola, A. I. & Druffel, E. R. M. Cycling of black carbon in the ocean. *Geophys. Res. Lett*. **43**, 10.1002/2016GL068574 (2016).

[15] Coppola, A. I. et al. Marked isotopic variability within and between the Amazon River and marine dissolved black carbon pools. *Nat. Commun*. **10**, 10.1038/s41467-019-11543-9 (2019). 10.1038/s41467-019-11543-9 PMC6728373 31488815

[16] Beaupré, S. R. The carbon isotopic composition of marine DOC. In (eds Hansell, D. A. & Carlson, C. A.) *Biogeochemistry of Marine Dissolved Organic Matter* pp. 335–364. (Elsevier, Oxford, 2015).

[17] Raymond, P. A. & Spencer, R. G. M. Riverine DOM. In (eds Hansell, D. A. & Carlson, C. A.) *Biogeochemistry of Marine Dissolved Organic Matter* pp. 509–533. (Elsevier, Oxford, 2015).

[18] Hansell DA, Carlson CA, Repeta DJ, Schlitzer R (2009). Dissolved organic matter in the ocean: a controversy stimulates new insights. Oceanography.

[19] Hedges JI, Keil RG, Benner R (1997). What happens to terrestrial organic matter in the ocean?. Org. Geochem..

[20] Druffel ERM, Williams PM, Bauer JE, Ertel JR (1992). Cycling of dissolved and particulate organic matter in the open ocean. J. Geophys. Res..

[21] Wagner, S. *et al* Online quantification and compound-specific stable isotopic analysis of black carbon in environmental matrices via liquid chromatography-isotope ratio mass spectrometry. *Limnol. Oceanogr.* **15**, 10.1002/lom3.10219 (2017).

[22] Ward ND (2015). The compositional evolution of dissolved and particulate organic matter along the lower Amazon River—Óbidos to the ocean. Mar. Chem..

[23] Spencer, R. G. M. et al. Origins, seasonality, and fluxes of organic matter in the Congo River. *Glob. Biogeochem. Cycles* **30**, 1105–1121 (2016).

[24] Johnston SE (2018). Flux and seasonality of dissolved organic matter from the Northern Dvina (Severnaya Dvina) River, Russia. J. Geophys. Res..

[25] Mann, P. J. et al. Controls on the composition and lability of dissolved organic matter in Siberia's Kolyma River basin. *J. Geophys. Res.* **117**, 10.1029/2011JG001798 (2012).

[26] Bianchi TS, Filley T, Dria K, Hatcher PG (2004). Temporal variability in sources of dissolved organic carbon in the lower Mississippi River. Geochim. Cosmochim. Acta.

[27] Dittmar T, Koch B, Hertkorn N, Kattner G (2008). A simple and efficient method for the solid-phase extraction of dissolved organic matter (SPE-DOM) from seawater. Limnol. Oceanogr..

[28] Dittmar T (2008). The molecular level determination of black carbon in marine dissolved organic matter. Org. Geochem..

[29] Kappenberg A, Blasing M, Lehndorff E, Amelung W (2016). Black carbon assessment using benzene polycarboxylic acids: Limitations for organic-rich matrices. Org. Geochem..

[30] Butman, D., Raymond, P. A., Butler, K. & Aiken, G. Relationships between Δ^14^C and the molecular quality of dissolved organic carbon in rivers draining the coast from the continuous United States. *Global Biogeochem. Cy*cles **26**, 10.1029/2012GB004361 (2012).

[31] Kohn MJ (2010). Carbon isotope compositions of terrestrial C3 plants as indicators of (paleo) ecology and (paleo) climate. Proc. Natl Acad. Sci. USA.

[32] Cerling TE (1997). Global vegetation change through the Miocene/Pliocene boundary. Nature.

[33] Foley, J. A., Kucharik, C. J., Twine, T. E., Coe, M. T., Donner, S. D. Land use, land cover, and climate change across the Mississippi basin: impacts on selected land and water resources. In (eds Defries, R. S., Asner, G. P. & Houghton, R. A.) *Ecosystems and Land Use Change*, *Volume 15* pp. 249–261 (American Geophysical Union, 2004).

[34] Spencer, R. G. M., Stubbins, A. & Gaillardet, J. Geochemistry of the Congo River, estuary and plume. In (eds Bianchi, T. S., Allison, M. A. & Cai, W. J.), *Biogeochemical Dynamics at Large River-Coastal Interfaces: Linkages with Global Climate Change* pp. 554–584. (Cambridge University Press, Cambridge, 2014).

[35] Holmes, R. M. et al. Climate change impacts on the hydrology and biogeochemistry of Arctic rivers. In (eds Goldman, C. R., Kumagai, M. & Robarts, R. D.), *Climatic Change and Global Warming of Inland Waters: Impacts and Mitigation for Ecosystems and Societies* pp. 3–26. (John Wiley, Chichester, 2013).

[36] Spencer RGM (2012). An initial investigation into the organic matter biogeochemistry of the Congo River. Geochim. Cosmochim. Acta.

[37] Moran MA (2016). Deciphering ocean carbon in a changing world. PNAS.

[38] Kaiser K, Benner R (2008). Major bacterial contribution to the ocean reservoir of detrital organic carbon and nitrogen. Limnol. Oceanogr..

[39] Dai A, Trenberth KE (2002). Estimates of freshwater discharge from continents: latitudinal and seasonal variations. J. Hydrometeorol..

[40] Hernes PJ, Benner R (2006). Terrigenous organic matter sources and reactivity in the North Atlantic Ocean and a comparison to the Arctic and Pacific Oceans. Mar. Chem..

[41] Hernes PJ, Benner R (2002). Transport and diagenesis of dissolved and particulate terrigenous organic matter in the North Pacific Ocean. Deep-Sea Res. Pt. I.

[42] Opsahl S, Benner R (1997). Distribution and cycling of terrigenous dissolved organic matter in the ocean. Nature.

[43] Ziolkowski, L. A. & Druffel, E. R. M. Aged black carbon identified in marine dissolved organic carbon. *Geophys. Res. Lett*. **37**, 10.1029/2010GL043963 (2010).

[44] Stubbins A, Niggemann J, Dittmar T (2012). Photo-lability of deep ocean dissolved black carbon. Biogeosci.

[45] Wagner S, Jaffé R (2015). Effect of photodegradation on molecular size distribution and quality of dissolved black carbon. Org. Geochem..

[46] Spencer, R. G. M. et al. Photochemical degradation of dissolved organic matter and dissolved lignin phenols from the Congo River. *J. Geophys. Res*. **114**, 10.1029/2009JG000968 (2009).

[47] Lalonde K, Vahatalo AV, Gelinas Y (2014). Revisiting the disappearance of terrestrial dissolved organic matter in the ocean: a δ^13^C study. Biogeosci.

[48] Benner R, Fogel ML, Sprague EK, Hodson RE (1987). Depletion of ^13^C in lignin and its implications for stable carbon isotope studies. Nature.

[49] Coppola, A. I., Ziolkowski, L. A., Masiello, C. A. & Druffel, E. R. M. Aged black carbon in marine sediments and sinking particles. *Geophys. Res. Lett*. **41**, 10.1002/2013GL059068 (2014).

[50] Bauer, J. E. Carbon Isotopic Composition of DOM. In: (eds Hansell, D. A., Carlson, C. A.), *Biogeochemistry of Marine Dissolved Organic Matter*. pp. 405–453. 10.1016/B978-012323841-2/50010-5 (Elsevier, 2002).

[51] Jiao N (2010). Microbial production of recalcitrant dissolved organic matter: long-term carbon storage in the global ocean. Nat. Rev. Microbiol..

[52] Dittmar, T. Reasons behind the long-term stability of dissolved organic matter. In (eds Hansell, D. A. & Carlson, C. A.), *Biogeochemistry of Marine Dissolved Organic Matter* pp. 369–388. (Elsevier, Oxford, 2015).

[53] Rossel, P. E. et al. Thermally altered marine dissolved organic matter in hydrothermal fluids. *Org. Geochem*. **110**, 10.1016/j.orggeochem.2017.05.003 (2017).

[54] Hawkes, J. A. et al. Efficient removal of recalcitrant deep-ocean dissolved organic matter during hydrothermal circulation. *Nat. Geosci*. **8**, 10.1038/NGEO2543 (2015).

[55] Dickens AF, Gelinas Y, Masiello CA, Wakeham S, Hedges JI (2004). Reburial of fossil organic carbon in marine sediments. Nature.

[56] Galy V, Peucker-Ehrenbrink B, Eglinton T (2015). Global carbon export from the terrestrial biosphere. Nature.

[57] Hindersmann B, Achten C (2017). Accelerated benzene polycarboxylic acid analysis by liquid chromatography–time-of-flight–mass spectrometry for the determination of petrogenic and pyrogenic carbon. J. Chromatogr. A.

[58] Chang Z (2018). Benzenevpolycarboxylic acid—a useful biomarker for condensed organic matter, but not for only pyrogenic black carbon. Sci. Tot. Environ..

[59] Ziolkowski LA, Chamberlin AR, Greaves J, Druffel ERM (2011). Quantification of black carbon in marine systems using the benzene polycarboxylic acid method: a mechanistic and yield study. Limnol. Oceanogr..

[60] Bao, H., Niggemann, J., Luo, L., Dittmar, T. & Kao, S.-J. Aerosols as a source of dissolved black carbon to the ocean. *Nat. Commun*. **8**, 10.1038/s41467-017-00437-3 (2017). 10.1038/s41467-017-00437-3 PMC5593878 28894096

[61] Louisse C, Penner JE, Walton JJ, Eddleman H, Cachier H (1996). A global three-dimensional model study of carbonaceous aerosols. J. Geophys. Res..

[62] Gustafsson O (2009). Brown clouds over South Asia: Biomass or fossil fuel combustion?. Science.

[63] Eglinton, T. I. et al. Composition, age, and provenance of organic matter in NW African dust over the Atlantic Ocean. *Geochem. Geophys. Geosyst*. **3**, 10.1029/2001GC000269 (2002).

[64] Zencak Z, Elmquist M, Gustafsson O (2007). Quantification and radiocarbon source apportionment of black carbon in atmospheric aerosols using the CTO-375 method. Atmos. Environ..

[65] Szidat, S. et al. Dominant impact of residential wood burning on particulate matter in Alpine valleys during winter. *Geophys. Res. Lett*. **34**, 10.1029/2006GL028325 (2007).

[66] Mouteva, G. O. et al. Using radiocarbon to constrain black and organic carbon aerosol sources in Salt Lake City. *J. Geophys. Res.* **122**, 9843–9857 10.1002/2017JD026519 (2017).

[67] Bird MI, Ascough P (2012). Isotopes in pyrogenic carbon: a review. Org. Geochem..

[68] Lohmann, R. et al. Fluxes of soot black carbon to South Atlantic sediments. *Glob. Biogeochem. Cycles* **23**, 10.1029/2008GB003253 (2009).

[69] Moriera-Turcq P, Seyler P, Guyot JL, Etcheber H (2003). Exportation of organic carbon from the Amazon River and its main tributaries. Hydrol. Process..

[70] Mayorga E (2005). Young organic matter as a source of carbon dioxide outgassing from Amazonian rivers. Nature.

[71] Stubbins, A. et al. Utilizing colored dissolved organic matter to derive dissolved black carbon export by arctic rivers. *Front. Earth Sci*. **3**, 10.3389/feart.2015.00063 (2015).

[72] Schlitzer, R. Ocean Data View, odv.awi.de (2018).

