# Supplementary material for: Isotopic composition of oceanic dissolved black carbon reveals non-riverine source
Source: Nat Commun. 2019 Nov 7;10:5064. doi: 10.1038/s41467-019-13111-7 (PMC6838092; doi:10.1038/s41467-019-13111-7)
Supplement: Supplementary file 2 — Reporting Summary [file 41467_2019_13111_MOESM2_ESM.pdf]

nature research

Corresponding author(s): Sasha Wagner

Last updated by author(s): Sep 2, 2019

Reporting Summary

Nature Research wishes to improve the reproducibility of the work that we publish. This form provides structure for consistency and transparency in reporting. For further information on Nature Research policies, see Authors & Referees and the Editorial Policy Checklist.

Statistics

For all statistical analyses, confirm that the following items are present in the figure legend, table legend, main text, or Methods section.

n/a	Confirmed	
☐	☒	The exact sample size (n) for each experimental group/condition, given as a discrete number and unit of measurement
☐	☒	A statement on whether measurements were taken from distinct samples or whether the same sample was measured repeatedly
☐	☒	The statistical test(s) used AND whether they are one- or two-sided<br>*Only common tests should be described solely by name; describe more complex techniques in the Methods section.*
☒	☐	A description of all covariates tested
☐	☒	A description of any assumptions or corrections, such as tests of normality and adjustment for multiple comparisons
☐	☒	A full description of the statistical parameters including central tendency (e.g. means) or other basic estimates (e.g. regression coefficient) AND variation (e.g. standard deviation) or associated estimates of uncertainty (e.g. confidence intervals)
☐	☒	For null hypothesis testing, the test statistic (e.g. F, t, r) with confidence intervals, effect sizes, degrees of freedom and P value noted<br>*Give P values as exact values whenever suitable.*
☒	☐	For Bayesian analysis, information on the choice of priors and Markov chain Monte Carlo settings
☒	☐	For hierarchical and complex designs, identification of the appropriate level for tests and full reporting of outcomes
☒	☐	Estimates of effect sizes (e.g. Cohen's d, Pearson's r), indicating how they were calculated

Our web collection on statistics for biologists contains articles on many of the points above.

Software and code

Policy information about availability of computer code

Data collection	DOC values were obtained via the Shimadzu TOC-Control V software, then exported to Excel for calibration against known standards. BPCA concentrations were determined using the Thermo Scientific Chromeleon Chromatography Data System software, then exported to Excel for calibration against known standards. Stable carbon isotopic values were obtained using the Thermo Scientific Isodat software package, then data were exported to Excel for calibration against known standards.
Data analysis	Statistical evaluations were performed using JMP or Excel.

For manuscripts utilizing custom algorithms or software that are central to the research but not yet described in published literature, software must be made available to editors/reviewers. We strongly encourage code deposition in a community repository (e.g. GitHub). See the Nature Research guidelines for submitting code & software for further information.

Data

Policy information about availability of data

All manuscripts must include a data availability statement. This statement should provide the following information, where applicable:

- Accession codes, unique identifiers, or web links for publicly available datasets
- A list of figures that have associated raw data
- A description of any restrictions on data availability

The authors declare that the data supporting the findings of this study are available within the paper and its supplementary information files. The source data underlying Figures 2, 3, and 4 are provided in Table 1, Table S1, and the Source Data file.

Field-specific reporting

Please select the one below that is the best fit for your research. If you are not sure, read the appropriate sections before making your selection.

☐ Life sciences ☐ Behavioural & social sciences ☒ Ecological, evolutionary & environmental sciences

For a reference copy of the document with all sections, see nature.com/documents/nr-reporting-summary-flat.pdf

Ecological, evolutionary & environmental sciences study design

All studies must disclose on these points even when the disclosure is negative.

Study description

In the current study, we used compound-specific δ13C to probe the persistence of riverine DBC in the open ocean within a globally relevant sample set. If rivers were not the primary source of DBC in the ocean, then we would expect the δ13C signature of oceanic DBC to be different from that of rivers. If the null hypothesis was upheld, then oceanic DBC would exhibit δ13C signatures similar to that of riverine DBC. To test this hypothesis, water samples were collected from five major world rivers (Amazon, Congo, Northern Dvina, Kolyma, and Mississippi) and from the Pacific and Atlantic Oceans.

Research sample

The sampled rivers deliver ~24% of freshwater discharge, ~17% of DOC and therefore ~17% of DBC to the oceans. The rivers vary in their DOC composition, land use, catchment area, geographical location, and climate, thereby capturing a wide range of potential terrestrial DBC sources and isotopic signatures. Ocean samples were chosen to capture globally representative water masses and gradients in bulk DOC quality and source.

Sampling strategy

All seawater samples were collected using Niskin bottles mounted to the CTD rosette. For each sample, ~10 L was passed, by gravity, through a pre-cleaned in-line capsule filter (Whatman Polycap; 0.2 µm) into acid-cleaned Nalgene fluorinated HDPE carboys and pre-combusted glass vials and acidified to pH 2 using HCl prior to solid phase extraction and DOC analysis.

All river samples were collected in situ using an oil-free pump equipped with a pre-cleaned, in-line capsule filter (Whatman Polycap, 0.2 µm) directly into acid-cleaned Nalgene bottles and pre-combusted glass vials. Samples were acidified to pH 2 using HCl prior to solid phase extraction and DOC analysis.

Data collection

S.W. measured, recorded, and performed all data analyses. DOC values were obtained via the Shimadzu TOC-Control V software, then exported to Excel for calibration against known standards. BPCA concentrations were determined using the Thermo Scientific Chromeleon Chromatography Data System software, then exported to Excel for calibration against known standards. Stable carbon isotopic values were obtained using the Thermo Scientific Isodat software package, then data were exported to Excel for calibration against known standards.

Timing and spatial scale

Pacific Ocean samples were collected from Station ALOHA (22.45°N, 158.00°W) during the Hawaii Ocean Time-series (HOT) 301 cruise aboard the R/V Ka`imikai-O-Kanaloa. Samples from surface and mesopelagic depths (5, 110, 765, and 1000 m) were obtained from two casts on April 17, 2018 and the deep water sample (3500 m) was collected on April 18, 2018. Atlantic Ocean samples were collected from Hydrostation S (31.67°N, 64.17°W) during the Bermuda Atlantic Time Series (BATS) 358 cruise aboard the R/V Atlantic Explorer. Water samples were collected along a depth profile (1, 70, 2000, and 3000 m) on April 8, 2019.

The Amazon River is the largest river on Earth, drains the world's largest tropical rainforest, and represents a major link in the transfer of carbon between land and sea. One surface water sample was collected from the Amazon River near Óbidos (Brazil; April 20, 2018; 1.92°S, 55.53°W). The Congo River, located in equatorial Africa, is the second largest river on Earth and was sampled from a site upstream of the cities of Kinshasa-Brazzaville on the main stem (DR Congo; 4.18°S, 15.21°E). Five samples were collected from the Congo River between August 2011 and April 2012, where discharge ranged from 23,740 to 54,120 m3 s-1, to account for potential hydrologically driven variations in DBC isotopic composition in a low latitude, subtropical river. The Northern Dvina River watershed is located in northwestern Russia, primarily drains low relief peatlands, is not underlain by permafrost, and is a major contributor to pan-Arctic terrestrial DOC fluxes. Four samples were collected from the Northern Dvina River in Arkhangelsk (64.55°N, 40.51°E) between October 2013 and May 2016, where discharge ranged from 637 to 13,380 m3 s-1, to capture isotopic variations in a high latitude river with extremely variable hydrology. The Kolyma River discharges into the Arctic Ocean and is a large, northern high-latitude river system that is, in contrast to the Northern Dvina, completely underlain by continuous permafrost. One sample was collected from the Kolyma River, upstream of the city of Cherskiy (August 30, 2015; 68.75°N, 161.29°E). The Mississippi River drainage basin spans ~40% of the conterminous United States and contains one of the most productive agricultural regions in the world. One sample was collected from the Belle Chase Ferry Landing on the Mississippi River, just south of New Orleans, LA (USA; February 23, 2016; 29.82°N, 90.00°W).

Data exclusions

N/A

Reproducibility

River samples were oxidized and analyzed in duplicate and ocean samples in triplicate for BPCA quantification and determination of DBC concentrations. River samples were analyzed in duplicate and ocean samples analyzed in triplicate for bulk SPE-DOC and BPCA-specific δ13C analysis.

Randomization

Samples were categorized as riverine or oceanic as described above.

Blinding

N/A

Did the study involve field work? ☒ Yes ☐ No

Field work, collection and transport

Field conditions	Field conditions relevant to the outcome of this study are included in the "Timing and Spatial Scale" section above.
Location	See "Timing and Spatial Scale" section above for details.
Access and import/export	Sampling and transport of samples were carried out in compliance with local, national, and international laws.
Disturbance	Samples were collected as part of ongoing research efforts in each location, therefore additional disturbance was minimized.

Reporting for specific materials, systems and methods

We require information from authors about some types of materials, experimental systems and methods used in many studies. Here, indicate whether each material, system or method listed is relevant to your study. If you are not sure if a list item applies to your research, read the appropriate section before selecting a response.

Materials & experimental systems

n/a	Involved in the study	
☒	☐	Antibodies
☒	☐	Eukaryotic cell lines
☒	☐	Palaeontology
☒	☐	Animals and other organisms
☒	☐	Human research participants
☒	☐	Clinical data

Methods

n/a	Involved in the study	
☒	☐	ChIP-seq
☒	☐	Flow cytometry
☒	☐	MRI-based neuroimaging
